# Hepatitis C Virus Saint Petersburg Variant Detection With Machine Learning Methods

**DOI:** 10.1002/jmv.70169

**Published:** 2025-02-17

**Authors:** Nurhan Arslan, Bernhard Reuter, Joachim Buech, Thomas Lengauer, Martin Obermeier, Rolf Kaiser, Nico Pfeifer

**Affiliations:** ^1^ Department of Computer Science, Methods in Medical Informatics University of Tuebingen Tübingen Germany; ^2^ Departments Computational Biology & Applied Algorithmics Max Planck Institute for Informatics Saarbruecken Germany; ^3^ DZIF, Deutsches Zentrum für Infektionsforschung, German Center for Infection Research Partner Site Bonn‐Cologne Cologne Germany; ^4^ Medical Center for Infectious Diseases Berlin Germany; ^5^ Laboratory for Viral Resistance Research, Institute of Virology University and University Clinics of Cologne Cologne Germany

**Keywords:** geno2pheno_[HCV]_, HCV 2k/1b variant, hepatitis C virus, machine learning, molecular epidemiology

## Abstract

Hepatitis C virus infection is a significant global health concern, affecting millions worldwide. Although direct‐acting antivirals achieve over 90% success rate, treatment failures still occur, particularly when pan‐genotypic DAAs are unavailable, and drugs need to be chosen based on the present HCV genotype. Genotyping tests can be misleading, especially in cases involving the 2k/1b recombinant variant. The 2k/1b variant was first discovered in Saint Petersburg in 2002 and is most commonly observed in Eastern European countries, including Russia, Georgia, and Ukraine. Due to migration, the 2k/1b variant has spread to Western Europe and other regions, potentially increasing HCV transmission and changing the virus's epidemiological landscape. The situation highlights the importance of molecular epidemiology in monitoring the spread of the 2k/1b variant. Accurate detection and characterization of the 2k/1b variant are crucial for an effective treatment if no pan‐genotypic DAAs are available. To address this need, machine learning models were developed to predict the 2k/1b variant based on 1b and 2k/1b sequence data from nonstructural proteins. They were integrated into the geno2pheno_[HCV]_ tool, providing physicians and researchers with an open‐access resource for determining HCV genotypes, including the 2k/1b variant.

**Abbreviations**
CV: cross validation
DAAs: direct‐acting antiviral agents
HCV: hepatitis C virus
ML: machine learning

## 1 Introduction

Hepacivirus hominis (hepatitis C virus, HCV) is a species in the genus *hepacivirus*, family *flaviviridae*. It is an important human pathogen causing severe health problems worldwide. It primarily spreads through blood and body fluids. The absolute number of HCV‐infected individuals is unclear. The World Health Organization (WHO) estimates approximately 58 million chronic HCV cases worldwide, with 1.8 million new infections each year. Chronic HCV infection can progress to cirrhosis, hepatocellular carcinoma, and liver failure, resulting in 500 000–700 000 deaths annually [1, 2]. Direct‐acting antivirals (DAAs), introduced in 2011, have become the most effective HCV treatment, achieving a 90% cure rate [3]. In 2015, the WHO aimed to reduce HCV incidence by 90% and mortality by 65% until 2030, followed by the success of DAAs [4]. However, considering the increase in the reported global HCV rates, many countries are falling short of the WHO's plan [5]. Multiple factors prevent the achievement of the WHO targets for 2030. A profound understanding of the epidemiology of HCV is required, which not only includes appropriate diagnostics to detect infections but also requires detailed information on the distribution of genotypes. Especially, but not only in resource‐limited settings, treatment strategies based on genotyping allow for more efficient treatments in terms of success and cost‐effectiveness [6].

HCV is a single‐stranded RNA virus and has seven genotypes and more than 90 subtypes. Furthermore, eight intra‐genotype recombinant variant forms have been observed [7, 8, 9]. Genetic recombination has a significant effect on viral diversity and also produces rare forms of intergenotypic recombinants [3, 8, 10].

The HCV genome encodes 10 proteins, each playing a different role in the viral life cycle. Of those, three proteins are structural, and six proteins are nonstructural, while the function of one protein located between the structural and nonstructural region of HCV, namely p7, is still not completely resolved. C, E1, and E2 are structural proteins responsible for receptor binding, viral entry, fusion, and viral capsid formation. They are followed by p7, which is suspected to perform ion‐channel activity [11], and NS2, NS3, NS4A, NS4B, NS5A, and NS5B, which are nonstructural proteins that coordinate various steps of viral replication such as RNA replication machinery, host innate immune response control, and viral assembly [12, 13, 14].

The effectiveness of HCV treatment is influenced by factors like the virus's genetic diversity, high mutation rate, rare recombination mechanism, and therapeutic approach [3, 12]. DAAs target nonstructural proteins, with three classes: NS3 protease inhibitors (polyprotein processing), NS5A inhibitors (replication and assembly), and NS5B polymerase inhibitors (RNA replication) [5, 12, 13]. The potency of some HCV drugs administered in various countries depends on the viral genotype, making it important to identify the specific sub‐genotype for better treatment outcomes. In 5%–10% of cases, resistance mutations or recombinant variants reduce DAA effectiveness [13, 15]. Reinfection in high‐risk groups complicates treatment, emphasizing the need for accurate genotyping and resistance detection across genotypes, subtypes, and recombinant variants [13]. Exact genotyping and detection of recombinant forms is the basis for molecular epidemiology to detect the distribution of the different HCV variants. An illustration provided in Supporting Information S1: Appendix A presents the HCV genome, with a focus on the 2k/1b variant, and outlines the targets of DAAs along with their associated inhibitors.

The first circulating recombinant form of HCV, the Saint Petersburg or 2k/1b variant, was discovered in 2002 in Saint Petersburg, Russia [16]. It combines genetic material from genotypes 2k and 1b, differing significantly from other known genotypes and creating a new strain [17]. Genetic divergence was caused by recombination between HCV genomes of different genotypes rather than by mutation [16, 17]. The 2k/1b variant is an intergenotype recombinant between subtype 2k and subtype 1b and has a 5’ genome region that is most closely related to subtype 2k and a 3’ genome region that is most closely related to the global epidemic subtype 1b, with a single recombination breakpoint located in the NS2 gene [18]. Among all HCV variants and recombinant forms, only the 2k/1b variant has been recognized as a notable health concern due to its potential treatment challenges [16].

Since its discovery, the 2k/1b variant has been detected in multiple locations, including Russia, France, Ireland, Greece, and Georgia [8, 16, 17]. A 2010 case report highlighted the importance of considering 2k/1b variant in diagnosis, treatment response, and its epidemiological spread [19]. Susser's 2017 study linked the increased prevalence of the 2k/1b variant to migration from the former Soviet Union, particularly to Germany and Israel [20]. The 2k/1b variant is also becoming more prevalent in countries such as Germany, Italy, the Netherlands, and Spain, as well as in the other countries bordering Russia [8, 16, 17, 20]. Georgia represents about 20% of HCV 2k/1b cases, compared to 3% globally, emphasizing its spread through migration [17]. Recent migration from Ukraine to Europe may further increase the prevalence of 2k/1b variant and the emergence of new mutations.

Most commercial assays focus on the 5’‐UTR or core region of the HCV genome to determine its genotype. These assays often detect genotype 2 (GT2), which requires a different treatment approach than genotype 1 (GT1) [20, 21]. The correct assignment is especially relevant when non‐pan‐genotypic drug combinations are used [20, 21]. The DAAs target nonstructural proteins NS3, NS5A, and NS5B. In the case of 2k/1b, those proteins descend from genotype 1b and should hence be treated according to this genotype [20, 22]. The false assignment of genotype 2 is due to the fact the recombination breakpoint is located in the NS2 region [23]. So assays that only test for specific mutations in regions before the breakpoint are missing the required information for correct genotyping. Therefore, standard genotyping may misclassify the 2k/1b variant due to limitations in analyzing partial viral genomes, as whole‐genome sequencing remains costly and less accessible viral genomes [24]. Yet this form of genotyping is widely used in resource‐limited settings.

Given the findings of related studies, it is necessary to characterize drug‐resistant HCV mutations and variants to develop effective treatment strategies and conduct global epidemiological research, especially in high‐prevalence, low‐ and middle‐income countries [8, 16, 17, 20]. Machine learning (ML) methods can be used to develop predictive models that identify HCV genotypes, subtypes, and relevant mutations and variants to better understand how resistance‐associated mutations and recombinant variants influence treatment outcomes before treatment or if treatment has failed. ML is increasingly applied in the medical field for disease diagnosis, personalized treatment, drug development, robotic surgery, and predictive analytics, particularly in classification tasks for diseases like cancer, liver disease, Alzheimer's, and COVID−19 [25, 26, 27, 28, 29].

Geno2pheno is a freely available web tool that serves to analyze and interpret drug resistance in HCV and HIV genotypes and subtypes using HCV and HIV sequence data [13, 25]. There are two ways to assess antiviral drug resistance: Rule‐based interpretation systems, which rely on knowledge from expert panels, and statistics‐driven systems, which use statistical models based on ML algorithms trained on clinical or virological data [25]. The developers of the geno2pheno_[HCV]_ tool highlighted that the incidence of infections with drug‐resistant HCV variants is expected to rise, leading to more frequent treatment failures [13]. The aim of this study is to predict the HCV 2k/1b variant using an ML system that employs binary support vector machine (SVM) classifiers trained on sequence data. SVMs, belonging to the supervised ML methods, are commonly used in medical classification problems and have shown effectiveness in various applications [30]. The prediction engine reported here was integrated into the geno2pheno_[HCV]_ web tool, which provides physicians and researchers with an open‐access prediction service that can be used to get recommendations for the best anticipated antiviral treatment. This study introduces an HCV 2k/1b variant prediction tool within geno2pheno_[HCV]_, enabling the determination of the correct sub‐genotype for epidemiological analyses of a given HCV variant.

## 2 Materials and Methods

**Figure 1:**
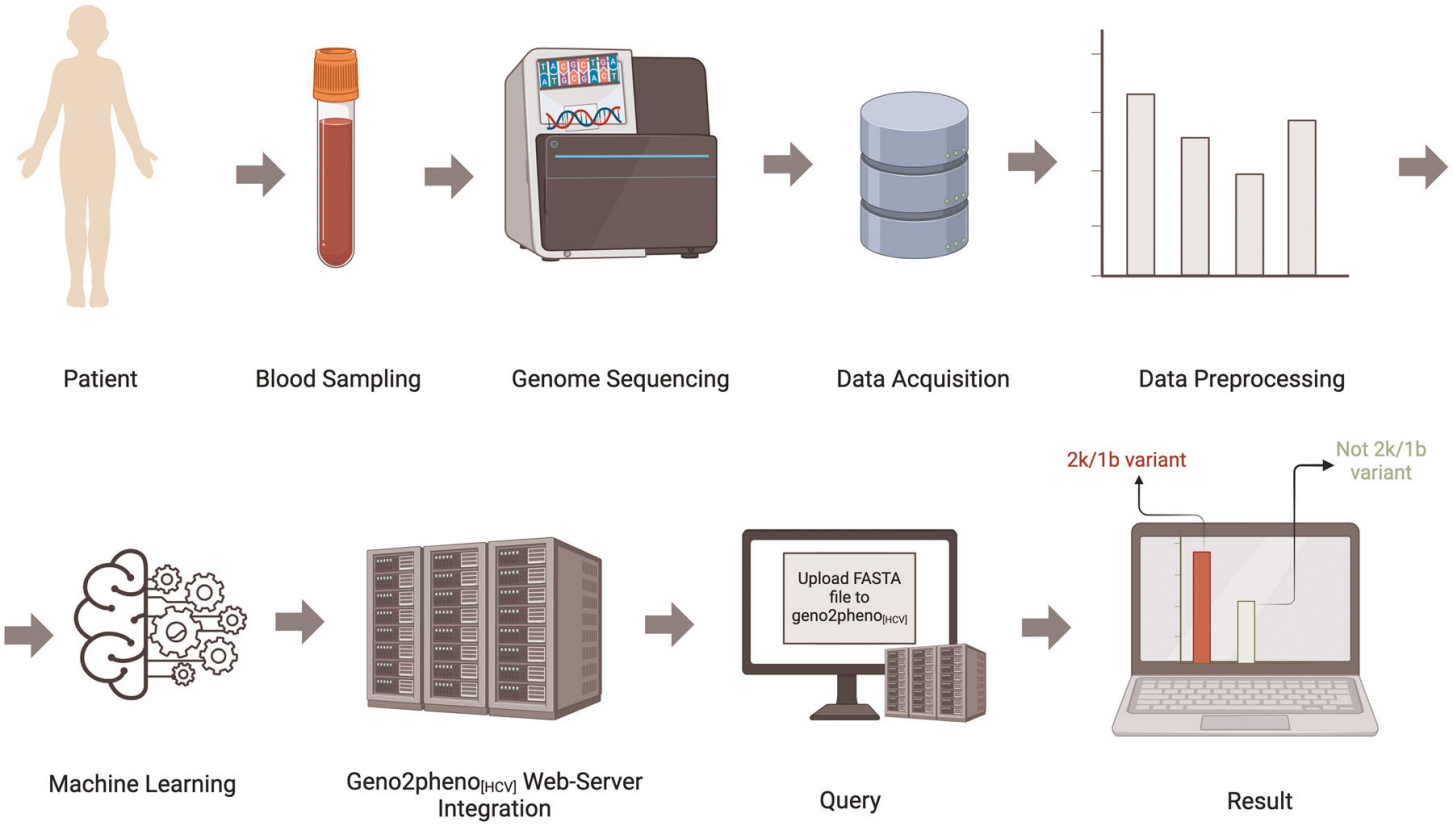
Flowchart of HCV 2k/1b variant prediction models [31]. The workflow begins with data collection, where blood samples are obtained from patients, and the HCV genome is sequenced. Data acquisition involves downloading the viral sequences from the NCBI nucleotide database. Data preprocessing includes down‐sampling, MSA, sample elimination, selection of sequence windows with the highest 2k/1b coverage, training data set construction, profile alignment, and encoding of class labels and features for model training. In the machine learning phase, predictive models are trained by using SVMs on the NS3, NS5A, and NS5B training data sets, followed by cross‐validation and testing of the models. From these models, NS3 and NS5A are integrated into the geno2pheno_[HCV]_ web tool. In the query process, users can upload FASTA files containing one or multiple sequences to determine whether the sequences are the 2k/1b variant. The results display the classification outcome, indicating the presence or absence of the 2k/1b variant in each uploaded sequence.

Three prediction models for the HCV 2k/1b variant were developed using supervised ML methods. These methods can be used to train a statistical model to predict labels for new samples using labeled training samples. Three separate binary classifiers were trained using linear and nonlinear SVMs to predict whether a certain variant is a 2k/1b variant based on the nucleotide sequences of the NS3, NS5A, and NS5B proteins, respectively. The models based on NS3 and NS5A were integrated into the geno2pheno_[HCV]_ web server. Each step in this process is illustrated in Figure 1. The implementation was performed using Python [32] and MATLAB [33].

### 2.1 HCV Nucleotide Sequence Data Set

Nucleotide sequences for the nonstructural HCV proteins NS3, NS5A, and NS5B were downloaded from the NCBI nucleotide database [34]. The searches were conducted using the terms “HCV and protein name and 1b and not 2k/1b” to extract genotype 1b sequences and the string “HCV and protein name and 2k/1b” to obtain 2k/1b sequences. These searches resulted in the acquisition of 15 703 1b and 76 2k/1b sequences for NS3, 25 196 1b and 61 2k/1b sequences for NS5A, and 12 601 1b and 66 2k/1b sequences for NS5B.

ML methods use past examples, known as training data, to learn and build a model that can predict outcomes for new, unseen data [35]. Whenever the unseen data are drawn from a distribution similar to that of the training data and if the training data harbor significant signals regarding the response, accurate predictions can be made for the unseen data [35]. In this study, the training data sets consist of nucleotide sequences of HCV nonstructural proteins, which are named NS3_NCBI_training, NS5A_NCBI_training, and NS5B_NCBI_training.

The training data sets include DNA bases (A, C, G, T) and DNA nucleotide codes defined by the International Union of Pure and Applied Chemistry (IUPAC) and used to identify an ambiguous nucleotide in a DNA sequence when the true nucleotide is a mixture of different nucleotides at a specific position.

### 2.2 Validation Data Sets

This study used two distinct validation data sets, labeled as 1b and 2k/1b validation data set, respectively, to evaluate 2k/1b prediction models. The 1b validation data sets are called NS3_NCBI_validation, NS5A_NCBI_validation, and NS5B_NCBI_validation, respectively, while 2k/1b validation data sets called NS3_MIB_validation, NS5A_MIB_validation, and NS5B_MIB_validation. Further details on these validation data sets are provided in Supporting Information S1: Appendices B and C.

### 2.3 Data Preprocessing

Data preprocessing was done in five steps: Data down‐sampling, multiple sequence alignment (MSA), data elimination, identification of sequence windows with maximum coverage based on the MSA results, and encoding of the class labels and features of the down‐sampled 1b and 2k/1b data sets for each target protein.

#### 2.3.1 Down‐Sampling

In the data sets NS3_NCBI_training, NS5A_NCBI_training, and NS5B_NCBI_training, there is a significant difference between the number of 2k/1b sequences compared to the number of 1b sequences. To address this imbalance and prevent the models from being biased toward the majority class (1b), the 1b sequences were down‐sampled in each training data set. Specifically, 1000 1b sequences were selected randomly from each data set to create a more balanced data set, improving the models’ ability to accurately predict the minority class (2k/1b) [36].

#### 2.3.2 Multiple Sequence Alignment

To determine the 2k/1b variant, it is necessary to identify relevant features in the nucleotide sequences that distinguish the 2k/1b variant from others. This process requires building an MSA. An MSA was performed for each target protein's 1b and 2k/1b sequences using the HCValign tool from Los Alamos National Laboratory. By using HCValign tool, the most highly conserved regions within the 1b and 2k/1b sequences for each protein were identified. The MSA settings configured in the HCValign tool included specifying the sequence type as mat peptide, performing the alignment using the MAFFT algorithm, applying codon alignment with a parameter value of 5, and selecting the reference sequence H77.

#### 2.3.3 Sample Elimination

In the first MSA results, some sequences had large gap regions, including “complete gap sequences” consisting entirely of gaps (‐). Gaps in an alignment indicate positions in which nucleotides are poorly aligned across sequences, and complete gap sequences provide no genetic information. Large gap regions suggest dissimilarity or insufficient information between aligned sequences. Therefore, all sequences with over 50% gaps, including complete gap sequences, were removed. After this filtering, the NS3_NCBI_training data set retained 886 1b sequences and all 76 2k/1b sequences. The NS5A_NCBI_training data set retained 676 1b sequences and 58 2k/1b sequences, while NS5B_NCBI_training kept 197 1b sequences and 56 2k/1b sequences.

#### 2.3.4 Sequence Windows With the Highest Coverage of 2k/1b

The remaining sequences in the MSA results still include gaps. The focus was placed on identifying specific regions, termed “windows” that demonstrated the highest alignment coverage for both genotype 1b and the 2k/1b variant across the MSA results for each target protein, as detailed in Supporting Information S1: Appendix D. A second MSA was performed, using these high‐coverage regions of the 1b genotype and 2k/1b variant, aiming to enhance the quality of the second MSA by focusing on sequences that were less noisy and more informative. In the second MSA, no gaps were observed for the 1b genotypes across all proteins.

While no gaps were found after the second MSA in the NS5A 2k/1b sequences, some of the NS3 and NS5B 2k/1b sequences exhibited gaps.

To comprehensively represent and accurately identify the 2k/1b variant, windows with the highest coverage for the 2k/1b variant were reselected following the second MSA. In our study, the window frames are given as nucleotide positions within the respective gene, rather than the whole genome. Specifically, for the NS3 MSA, the window covered positions 1–369, while for the NS5B MSA, it spanned positions 8 to 204. This method enhanced alignment quality, ensuring that both the 1b genotype and the 2k/1b variant were well‐represented in the data set.

#### 2.3.5 Profile Alignment

Profiles for the 1b and 2k/1b sequences were generated by performing an MSA for each target protein. From these MSA results, the sequences’ regions with the highest coverage for the genotype 1b and the 2k/1b variant were identified, as described in Sections 2.3.2 and 2.3.4. This approach allowed us to create accurate and representative profiles for the 1b genotype and the 2k/1b variant. A profile alignment was performed for each test sequence based on these profiles using the MUSCLE alignment program in the BioPython library [37]. During the profile alignment, the profiles remained unchanged, but the test sequences were aligned based on the intact profiles [37].

#### 2.3.6 Encoding Class Labels and Features

Nucleotide sequences were encoded using one‐hot encoding with each nucleotide represented by a vector corresponding to the alphabet (A, C, G, T, ‐). The vector has a 1 at the nucleotide position and 0 s elsewhere. For IUPAC codes, which represent ambiguous bases such as R (A or G) and K (G or T), the vector has fractional values, such as R being [0.5 0 0.5 0 0] and K being [0 0 0.5 0.5 0]. Class labels are encoded as 0 for 1b (negative) and 1 for 2k/1b (positive).

### 2.4 HCV 2k/1b Variant Prediction Models

The HCV 2k/1b prediction problem was formulated as a classification problem within the context of supervised learning in ML. In such tasks, a prediction may fall into one of two (or several, more generally) discrete classes, such as type A or type B, or susceptible versus resistant. Supervised learning involves constructing a predictive model based on a training data set consisting of (feature, label) pairs, enabling the prediction of labels for new data. SVMs are commonly used for small that effectively separate data points into distinct classes within a high‐dimensional Euclidean space.

In many real‐world data sets, linear decision boundaries between classes are not appropriate. However, SVMs can address nonlinear classification problems through the use of kernel functions, a powerful concept in ML. A kernel function effectively maps data into usually a higher‐dimensional Euclidean space, where a linear separation may become possible. The polynomial and Gaussian RBF kernels are among the most commonly used for tabular data. The decision boundary of a nonlinear SVM is a nonlinear hypersurface.

Three prediction models for the HCV 2k/1b variant were developed using three specified protein data sets. For each target protein, an SVM with a polynomial kernel was trained on the respective training data set to classify each sample as either a 1b or 2k/1b variant.

## 3 Cross Validation (CV)

Ten runs of five‐fold CV were performed, splitting the data set into training and test sets, with each data point assigned either to the training data set or the test data set but never to both. This repeated process ensures reliable performance estimates and can be used to determine optimal hyperparameters. Polynomial kernel parameters for SVM models were tuned by selecting polynomial degrees within the range of 1–5 with increments of 1 and cost parameters within the range of 10^–5^–10^1^ with increments of 10.

## 4 Results

The following section presents results for all HCV 2k/1b prediction models developed through grid search, CV, and retraining models. Model performance was further evaluated using patient‐derived 2k/1b samples in the NS3_MIB_validation and NS5A_MIB_validation data sets, as well as 1b sequences from NCBI's nucleotide database, referred to as NS3_NCBI_validation and NS5A_NCBI_validation data sets.

In binary classification, model performance is evaluated using metrics such as recall, precision, F1 score, and accuracy. These metrics rely on true positives (TP), false positives (FP), true negatives (TN), and false negatives (FN), which describe the outcomes of a model's predictions compared to the actual class labels.

(1) $$\text{Accuracy }=\frac{\text{TP}+\text{TN}}{\text{TP}+\text{FN}+\text{TN}+\text{FP}}$$

to medium‐sized data sets. A linear SVM classifies data by determining the optimal hyperplane, a linear decision boundary

(2) $$\text{Precision }=\frac{\text{TP}}{\text{TP}+\text{FP}}$$

(3) $$\text{Recall }\left(\text{TPR}\right)=\frac{\text{TP}}{\text{TP}+\text{FN}}$$

(4) $$\text{FPR }=\frac{\text{TN}}{\text{TN}+\text{FP}}$$

A reasonable explanation for this outcome is the higher amount of conserved motifs in the NS5B region than NS3 and NS5A, resulting in lesser evolutionary drift from the ancestral 1b in this region.

Receiver operating characteristic (ROC) curves plot the TPR against the FPR as the decision threshold varies. The area under the ROC curve (AUC or AUROC) is a quantitative measure of the model's ability to distinguish between positive and negative classes. Additional details are available in Supporting Information S1: Appendix C.

SVMs with a polynomial kernel have performed well in various medical classification problems, including diagnosing diabetes, cardiovascular diseases, cancer, and neurological disorders [38, 39]. Therefore, an SVM was trained for each protein model using all possible combinations of degrees and cost parameters as described above, conducting 10 runs of a five‐fold CV. A permutational approach was utilized to systematically and randomly select 250 1b sequences per iteration to train NS3 and NS5A models. This approach ensured diverse and representative sampling from the preprocessed NS3_NCBI_training and NS5A_NCBI_training data sets containing 886 and 676 1b sequences, respectively. Since only 197 sequences remained after downsampling, no sub‐sampling of the 2k/1b sequences was needed. Thus, the complete preprocessed NS5B_NCBI_training data set was used during training. In the third step, each prediction model's estimated average AUC performance was calculated. The final step involved determining which combination of hyperparameter values would provide the best performance results for each of the target protein models. The prediction models were then retrained with optimal hyperparameters on the complete training data set.

Based on 10 runs of 5‐fold CV, the performance of SVMs trained on the NS3_NCBI_training (*N* = *N*_1*b*_ + *N*_2*k*/1*b*_ = 250 + 76 = 326), NS5A_NCBI_training (*N* = *N*_1*b*_ + *N*_2*k*/1*b*_ = 250 + 58 = 308), and NS5B_NCBI_training (*N* = *N*_1*b*_ + *N*_2*k*/1*b*_ = 197 + 56 = 253) data sets was compared. As shown in Table 1, the NS3 HCV 2k/1b variant prediction model has the highest AUC of 0.7279, followed by the NS5A HCV 2k/1b variant prediction model with 0.6292 AUC. The NS5B HCV 2k/1b variant prediction model showed the lowest mean prediction performance among all models evaluated.

**Table 1:** Summary of model performance: The table presents the mean and standard deviation (stdev) of AUC scores for 2k/1b prediction models based on NS3, NS5A, and NS5B.

	NS3	NS5A	NS5B
mean	0.7279	0.6292	0.5968
stdev	0.0032	0.0042	0.0077

### 4.1 Findings From the Test Data

**Figure 2:**
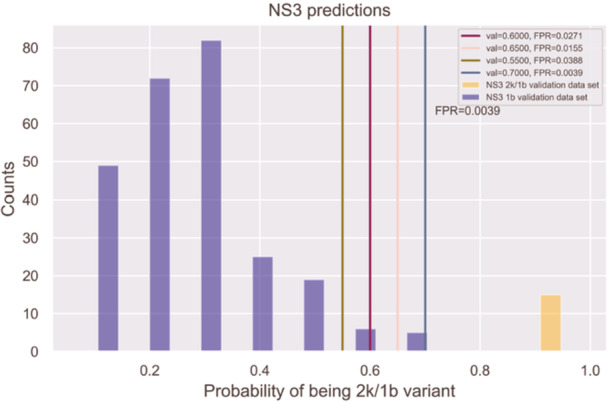
NS3 1b genotype and 2k/1b variant test results: The purple bars represent sequences identified as the 1b genotype, while the orange bars represent those identified as the 2k/1b variant. The x‐axis displays the probability of a sequence being the 2k/1b variant, and the y‐axis indicates the number of sequences identified at each probability level. Vertical lines in green, dark purple, pink, and blue mark thresholds of 0.55, 0.6, 0.65, and 0.7, respectively, representing the FPR for the 2k/1b variant at each threshold.

Figure 2 illustrates the results of testing the NS3 2k/1b prediction model on 258 1b samples from the NS3_NCBI_validation data set and 15 2k/1b samples from the NS3_MIB_validation data set. 2k/1b prediction probabilities ranged from 0.96 to 0.99 for the NS3 validation samples.

**Figure 3:**
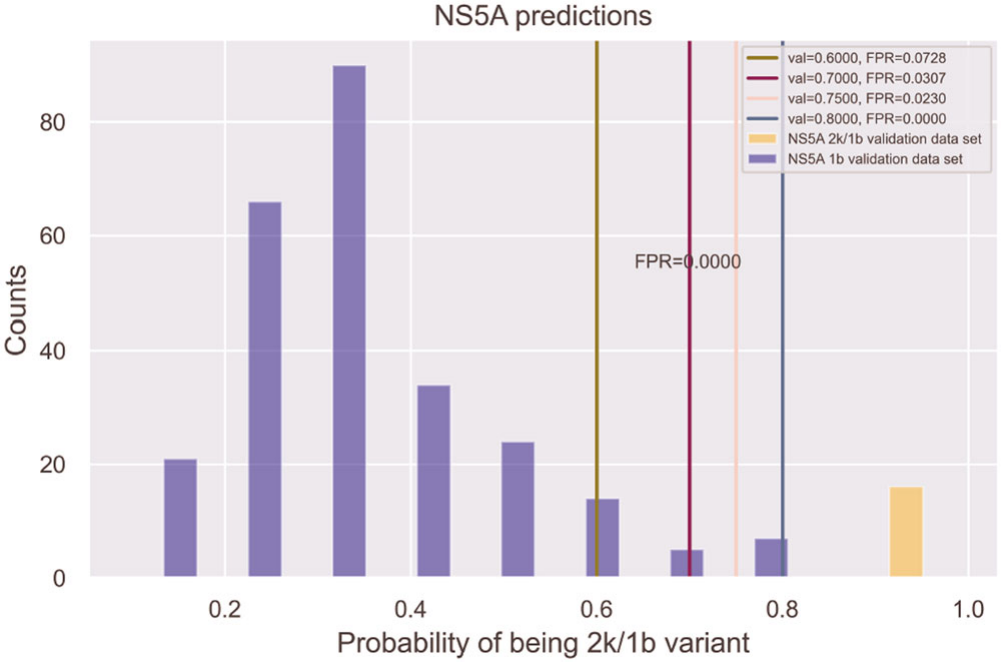
NS5A 1b genotype and 2k/1b variant test results: The purple bars represent sequences identified as the 1b genotype, while the orange bars represent those identified as the 2k/1b variant. The x‐axis displays the probability of a sequence being the 2k/1b variant, and the y‐axis indicates the number of sequences identified at each probability level. Vertical lines in green, dark purple, pink, and blue mark thresholds of 0.6, 0.7, 0.75, and 0.8, respectively, representing the FPR for the 2k/1b variant at each threshold.

Figure 3 illustrates the results of testing the NS5A 2k/1b prediction model on 258 1b samples from the NS5A_NCBI_validation data set and 16 2k/1b samples from the NS5A_MIB_validation data set. The prediction probabilities of the NS5A 2k/1b model spanned from 0.92 to 0.99 on the NS5A validation samples.

The NS3 and NS5A prediction models showed that all 2k/1b sequences from the NS5A_MIB_validation and NS3_MIB_validation data sets were detected without errors. The output of the prediction models consists of probabilities instead of just binary predictions, reflecting the probability of a sample being classified as a 2k/1b variant. In both NS3 and NS5A models, a prediction probability threshold of 0.90, which clearly distinguished 1b from 2k/1b sequences, was determined. The objective is to establish thresholds that clearly distinguish between 1b and 2k/1b sequences in both the NS3 and NS5A models. As noted, the prediction models successfully identified all 2k/1b sequences from both NS5A_MIB_validation and NS3_MIB_validation data sets without any misses when using thresholds of 0.9 or lower. While a threshold of 0.8 could reduce FP (where 1b is misclassified as 2k/1b), lower thresholds, such as 0.7 or 0.5, might be better if capturing all 2k/1b samples is the priority. Ultimately, it was decided to use a threshold of 0.9 to minimize the possibility of FP observations. This indicates that the clinicians prioritize reducing FP over capturing every possible 2k/1b sequence.

**Figure 4:**
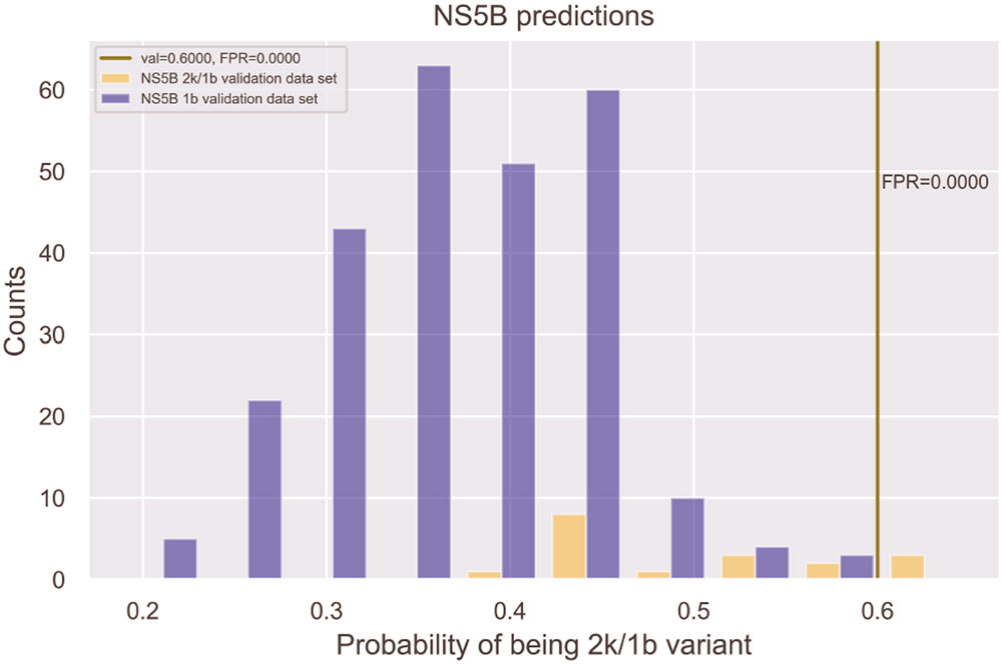
NS5B 1b genotype and 2k/1b variant test results: The purple bars represent sequences identified as the 1b genotype, while the orange bars represent those identified as the 2k/1b variant. The x‐axis displays the probability of a sequence being the 2k/1b variant, and the y‐axis indicates the number of sequences identified at each probability level. The vertical green line shows a threshold of 0.6, highlighting the FPR at this threshold.

The HCV 2k/1b prediction model for the NS5B protein had poor performance close to random prediction on the test data set compared to the NS3 and NS5A prediction models, as illustrated in Figure 4. As already stated above, a plausible explanation for this outcome is the higher amount of conserved motifs in the NS5B region as compared to NS3 and NS5A, resulting in less evolutionary drift from the ancestral 1b in this region [40, 41]. Thus, only the NS3 and NS5A prediction models were included in the prediction server.

### 4.2 Integration

The 2k/1b prediction models for the NS3 and NS5A proteins were integrated into the geno2pheno_[HCV]_ web server and are available at https://hcv.geno2pheno.org. Within this platform, users can submit their HCV FASTA sequences to the web server and receive an analysis indicating whether each antiviral medication's corresponding viruses are resistant or susceptible to the respective drug. If a sequence has a probability greater than 0.9 of being 2k/1b, a warning is displayed that the variant might be a 2k/1b variant.

## 5 Discussion

In this study, supervised ML was employed to predict the HCV 2k/1b variant, a recombinant strain resistant to treatment. Three prediction models were developed, each based on one of the key nonstructural proteins, NS3, NS5A, and NS5B, primary targets for DAAs. Training each model on protein‐specific sequence data allowed us to evaluate how reliably each model can predict the HCV 2k/1b variant.

Geno2pheno_[HCV]_ is the first web‐based and freely accessible server for the detailed analysis of HCV sequence data, focusing on the NS3, NS5A, and NS5B regions [13]. This tool aligns the input sequences, identifies the corresponding genomic regions, provides a detailed sub‐genotyping of HCV, and analyzes DAA‐ susceptibility for each drug target.

There are other tools for analyzing HCV sequence data, each of which has its own strengths and limitations. For example, VirVarSeq is open‐source software that detects codon‐level variants and specifically targets low‐frequency variants [42]. It is helpful in detecting minor viral populations, while it might have limitations to capture the full diversity of the virus. DiversiTools is a computational tool that analyzes mutation frequencies in haploid populations [43]. geno2pheno_[ngs‐freq]_ is a free, publicly accessible web server designed for the rapid genotypic interpretation of viral drug resistance in HIV‐1 and HCV NGS samples [25]. HCV‐GLUE is a bioinformatics tool for analyzing HCV sequencing data that provides genotype and subtype classification, drug resistance interpretation, genome visualization, and phylogenetic placement for NS3, NS5A, and NS5B [44, 45]. None of these tools are capable of analyzing or predicting the 2k/1b variant.

Recent progress with pan‐genotypic DAAs made HCV treatment less dependent on identifying specific genotypes [46]. The HCV guidelines of the American Association for the Study of Liver Disease recommend the pan‐genotypic treatment options Glecaprevir/Pibrentasvir or Sofosbuvir/Velpatasvir for the treatment of naive patients with genotype 2 infection [47]. In genotype 1b infections, the range of treatment options can be extended to Sofosbuvir/Ledipasvir and Grazoprevir/Elbasvir, which might be easily accessible and less cost‐intensive [17, 46]. Additionally, routine HCV genotyping does not typically include screening for recombinant strains, like the HCV 2k/1b variant, but rather limits the analysis to the core gene [21]. This oversight may result in the HCV 2k/1b variant being misclassified as genotype 2 (GT2), potentially leading to less effective treatment choices [20, 21]. Additionally, it can contribute to underdiagnosis, with its epidemiological studies remaining largely unexplored [21]. Addressing this issue requires more comprehensive techniques, such as whole‐genome or deep sequencing to identify breakpoints or developing affordable, cost‐effective precise tools specifically to detect the HCV 2k/1b variant [17, 21]. In resource‐limited settings, these sequencing methods are often impractical because they are expensive and time‐consuming, and pan‐genotypic DAAs remain largely inaccessible for similar reasons [21, 48]. As these regions also account for over 50% of global HCV cases, effective and accessible diagnostic and treatment strategies are especially needed [46].

This study introduces the first statistics‐driven prediction system for detecting the HCV 2k/1b variant. The system's results on test data confirm that accurately predicting the HCV 2k/1b variant is feasible using only the NS3 and NS5A nonstructural proteins without the need for more resource‐intensive whole genome or deep sequencing. These techniques are still needed in cases where the exact recombination breakpoint has to be identified. This is a systematic limitation of inferring recombination events from other parts of the genome without proper sequence data from the breakpoint region.

## 6 Conclusions

The freely accessible geno2pheno_[HCV]_ web tool promotes transparency, collaboration, and ongoing scientific contributions. It is particularly valuable in resource‐limited regions where whole‐genome sequencing and pan‐genotypic treatments are unaffordable or not feasible, enabling optimal treatment decisions for genotype‐specific DAA therapies using only NS3 and NS5A proteins in 2k/1b cases. The geno2pheno_[HCV]_ web tool can support epidemiological analysis, providing insights into 2k/1b prevalence, progression, and migration. These insights are vital for public health policy considerations. Considering the high prevalence of HCV 2k/1b in Eastern Europe and the potential spread through Ukrainian migration, epidemiological studies are essential for monitoring its trajectory. Continuous enhancement of prediction models will progress with the availability of more 2k/1b sequences in the future.

## Author Contributions

Nurhan Arslan, Nico Pfeifer, Rolf Kaiser, and Bernhard Reuter conceptualized the study. Nurhan Arslan preprocessed the data and implemented the ML models. Nurhan Arslan, Nico Pfeifer, Rolf Kaiser, Martin Obermeier, and Thomas Lengauer interpreted the results. Martin Obermeier collected the validation data. Joachim Buech integrated the study into the geno2pheno_[HCV]_ web server. Nurhan Arslan and Bernhard Reuter wrote the paper with support from the other authors. All authors reviewed and approved the final version of the manuscript.

## Conflicts of Interest

The authors declare no conflicts of interest.

## Supporting information

Supporting information.

Supporting information.

## Data Availability

Validation data and source code are available upon reasonable request from Martin Obermeier (validation data) MIB and Nurhan Arslan (source code).
