# Supplementary material for: Hepatitis C Virus Saint Petersburg Variant Detection With Machine Learning Methods
Source: J Med Virol. 2025 Feb 17;97(2):e70169. doi: 10.1002/jmv.70169 (PMC11831414; doi:10.1002/jmv.70169)
Supplement: Supplementary file 1 — Supporting information. [file JMV-97-e70169-s002.pdf]

☐

APPENDIX

A HCV GENOME AND DAAS TARGETS

Optimal HCV treatment requires accurate genotyping and subtyping, but standard assays, while effective for distinguishing HCV 1a and 1b, have limitations with genotype 2 subtyping. Most assays only analyze the 5'UTR or core region in non-genotype 1 samples, making them unable to detect chimeric HCV strains [1,2]. Using next-generation sequencing (NGS) affords examining the HCV NS3, NS5A, and NS5B genes, offering the advantage of detecting mixed infections and estimating their ratios, though it still cannot identify the 2k/1b variant strains [1].

Treatment for HCV genotype 2 is inadequate for cases involving the 2k/1b variant [1,3]. Though pan-genotypic DAAs can address genotype-specific treatment challenges, their high cost limits access. Six countries, China, Pakistan, Nigeria, Egypt, India, and Russia, comprise over half of global HCV cases [4]. Until affordable pan-genotypic treatments are more widely available, identifying recombinant strains can help guide treatment choices [1,3]. In high-prevalence regions, detecting the 2k/1b variant may also enhance antiviral response rates by using an optimal therapy, contributing to efforts to eliminate HCV worldwide [3].

B 1B VALIDATION DATASET

The following section describes the process for selecting 1b sequences of each protein to construct validation datasets for evaluating 2k/1b prediction models. The composition of the core proteins differs between the 2k/1b variant and the genotype 1b. The core protein of the 2k/1b variant contains the 2k segment, while the core protein of the genotype 1b contains the 1b segment. To avoid the risk of mislabeling 2k/1b sequences as 1b, the quality of sequences labeled as 1b in the validation dataset was improved. To achieve this, the core proteins in 1b sequences were compared with 1b and 2k/1b reference sequences. Initially, 296 1b sequences were retrieved from the NCBI nucleotide database, selecting those that met the criteria of being 1b and containing core protein sequences, i.e., NS3, NS5A, and NS5B, from the NCBI nucleotide database. The accession numbers of the 1b and 2k reference sequences were obtained from the Los Alamos HCV DB [9].

Pairwise alignments were performed between each core protein in the 1b sequences and the 1b and 2k/1b reference sequences, respectively. Following these alignments, sequence similarities were compared by calculating the difference between the alignment scores with the 1b and 2k/1b references. A negative difference indicated a greater similarity to the 2k/1b reference than the 1b reference. These calculations identified 258 sequences with positive differences, confirming their similarity to the 1b reference, while 38 sequences with negative differences showed greater similarity to the 2k/1b reference. This process ensured that the selected 1b sequences were accurately identified and not mislabeled as 2k/1b. The resulting 1b validation datasets were called NS3_NCBI_validation, NS5A_NCBI_validation, and NS5B_NCBI_validation, respectively.

C 2K/1B VALIDATION DATASET

In total, 49 additional HCV 2k/1b variant sequences were obtained from the MVZ MIB (Medizinisches Infektiologiezentrum Berlin) AG lab, encompassing three target proteins: NS3 (15 sequences), NS5A (16 sequences), and NS5B (18 sequences). In the same manner, as described above, these sequences were used to generate 2k/1b validation datasets.

D DIFFERENCES BETWEEN SEQUENCES DOWNLOADED FROM THE NCBI NUCLEOTIDE DATABASE IN 2020 AND 2024 AND THEIR GENBANK ACCESSION NUMBERS IN THE CURRENT NCBI NUCLEOTIDE DATABASE

The training dataset of each target protein was downloaded in 2020. Therefore, to determine whether any 2k/1b sequences of the target proteins had been updated in the NCBI nucleotide DB, the 2k/1b sequences from the NCBI nucleotide DB were recently downloaded again. Then, the 2k/1b sequences downloaded in 2020 were compared with those downloaded in 2024. Some sequences in the 2020 2k/1b dataset that were absent in the 2024 2k/1b dataset were identified. To gather more information about these sequences, the NCBI nucleotide database was searched using their accession numbers, and they were found. An attempt was made to understand why they could not be found using the original filter criteria outlined above. It was found that the "/product" part of the "Features" section was updated and included only "polyprotein" but no information about the names of non-structural proteins. Thus, filtering for NS3, NS5A, and NS5B did not deliver those sequences anymore. On the other hand, if one clicks on "/protein_id", one will still be able to access all information related to non-structural proteins. A list of sequence accession numbers found in 2020 but absent in 2024 when searching the NCBI nucleotide DB using the filter criteria above is presented below. These sequences were downloaded using their accession numbers from the NCBI nucleotide DB in 2024. To ensure that there have been no changes at the sequence level, those sequences were compared with the 2k/1b sequences downloaded in 2020. In doing so, no changes were observed at the sequence level for all sequences compared. It is possible to download related regions from different non-structural proteins for the accession numbers given here by first clicking the CDS or coded sequences section of the features section and then following the instructions above.

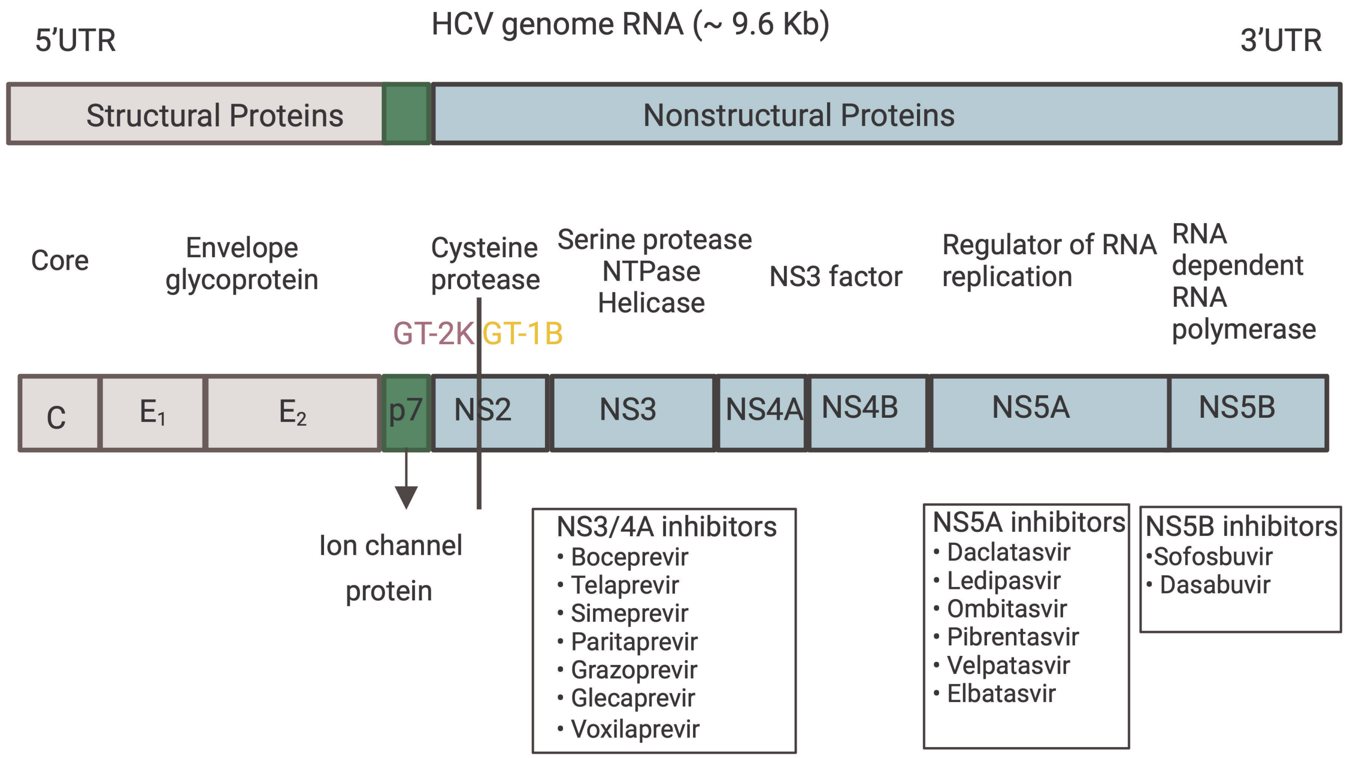

FIGURE A1 Illustration of the HCV RNA Genome, 2k/1b Variant, and DAA Targets and Drugs[5]. The illustration depicts the HCV genome with the 2k/1b variant. The HCV genome is composed of a single open reading frame encoding a polyprotein approximately 3,000 amino acids long. The genome is organized into two primary regions: the N-terminal region, which encodes structural proteins such as the core (C) protein and envelope proteins (E1 and E2), and the C-terminal region, which contains nonstructural proteins, colored in gray, involved in viral replication. The nonstructural proteins include NS2, NS3, NS4A, NS4B, NS5A, and NS5B depicted in blue. p7 is depicted in green[6,7]. DAAs specifically target the NS3 protease, NS5A protein, and NS5B polymerase, which are critical for the virus's lifecycle. The illustration highlights these target proteins alongside the corresponding inhibitors developed to neutralize them. Adapted from references[2,6,7,8].

1. HQ537005.1
2. HQ537006.1
3. JX227952.1
4. KM102765.1
5. KM102768.1
6. KM102769.1
7. KM102770.1
8. KM495736.1

E SEQUENCE WINDOWS WITH THE LARGEST COVERAGE OF 1B AND 2K/1B

The table below shows the window determined after the first MSA.

TABLE E1 Selected Sequence Windows After 1st MSA: The table illustrates the sequence windows for NS3, NS5A, and NS5B following the initial MSA.

	1b	2k/1b
**NS3**	10-528	10-372
**NS5A**	75-295	75-295
**NS5B**	746-942	740-946

F EVALUATION METRICS

In binary classification, the model's predictions can result in four possible outcomes: True Positive (TP), False Positive (FP), True Negative (TN), and False Negative (FN). These terms are used to compare the predicted class labels against the actual class labels in a dataset. A TP refers to an instance where the model correctly identifies a positive class, while an FP occurs

when the model incorrectly labels a negative instance as positive. Conversely, a TN is when the model accurately predicts a negative class and an FN arises when the model fails to identify a positive instance, incorrectly predicting it as negative. These outcomes form the basis for evaluating a model's classification performance. Those terms represent a number as a number of TPs, TNs, FPs, and FNs. These outcomes form the basis for several key metrics used to evaluate a model's performance, including recall, precision, F1 score, and accuracy.

Accuracy represents the overall correctness of the model's predictions, considering both true positives and true negatives, as shown in Equation 1. Precision indicates how many of the positive predictions were actually correct among all predicted positives, as shown in Equation 2. In the literature, the terms recall, sensitivity and True Positive Rate (TPR) are frequently used interchangeably, as they all describe the model's ability to correctly identify all actual positive labels, as shown in Equation 3. It is essential to select metrics that are appropriate for the application. False Positive Rate (FPR) measures the percentage of false positives against all positive predictions, as shown in Equation 4. The choice of metric depends on the specific application, as different metrics emphasize different aspects of performance.

REFERENCES

1. Susser S, Dietz J, Schlevogt B, et al. Origin, prevalence and response to therapy of hepatitis C virus genotype 2k/1b chimeras. *Journal of hepatology.* 2017;67(4):680–686.
2. Knops E, Heger E, Koenig C, et al. Accurate hepatitis C virus genotyping and selection of optimal therapy: lessons from a St Petersburg strain infection. *Clinical Microbiology and Infection.* 2018;24(4):440–441.
3. Zakalashvili M, Zarkua J, Gish RG, et al. Assessment of treatment options for patients with hepatitis C virus recombinant form 2k/1b. *Hepatology Research.* 2021;51(2):156–165.
4. Hézode C. Pan-genotypic treatment regimens for hepatitis C virus: Advantages and disadvantages in high-and low-income regions. *Journal of viral hepatitis.* 2017;24(2):92–101.
5. Arslan N. HCV_genome. Created in BioRender. https://BioRender.com/q43r113; 2024. Accessed: 2024-12-16.
6. Atoom AM, Taylor NG, Russell RS. The elusive function of the hepatitis C virus p7 protein. *Virology.* 2014;462:377–387.
7. Madan V, Bartenschlager R. Structural and functional properties of the hepatitis C virus p7 viroporin. *Viruses.* 2015;7(8):4461–4481.
8. Manns MP, Maasoumy B. Breakthroughs in hepatitis C research: from discovery to cure. *Nature reviews Gastroenterology & hepatology.* 2022;19(8):533–550.
9. Kuiken C, Yusim K, Boykin L, Richardson R. The Los Alamos hepatitis C sequence database. *Bioinformatics.* 2005;21(3):379–384.
