# Supplementary material for: Hepatitis C Virus Saint Petersburg Variant Detection With Machine Learning Methods
Source: J Med Virol. 2025 Feb 17;97(2):e70169. doi: 10.1002/jmv.70169 (PMC11831414; doi:10.1002/jmv.70169)
Supplement: Supplementary file 2 — Supporting information. [file JMV-97-e70169-s001.pdf]

Table 1: Selected Sequence Windows After 1st MSA: The table illustrates the sequence windows for NS3, NS5A, and NS5B following the initial MSA.

	**1b**	**2k/1b**
**NS3**	10-528	10-372
**NS5A**	75-295	75-295
**NS5B**	746-942	740-946
